# Philadelphia Academy of Surgery

**Published:** 1895-11

**Authors:** 


### Editorial.

Dr. Robert Battey, whose death was prematurely recorded in the medical press some months ago, died at his residence near Rome, Ga., November 8th, 1895.

Dr. Battey was born in Richmond county, Ga., November 26th, 1828. He received his education in local schools and at Phillip's Academy, Andover, Massachusetts. In early life, he was engaged in the drug business, but abandoned it for the study of medicine and graduated from the Jefferson Medical College, Philadelphia, in 1857. He soon after located in Rome, Ga., and built up a large general practice. In April, 1872, he performed the operation, now known by his name of, "normal" ovariotomy, the principle of which was to determine a premature change of life in women for the relief of menstrual neuroses. The operation was bitterly opposed and censured, but subsequently became popular. Of late, however, there has been a decided reaction against it.

In 1882, Dr. Battey established an infirmary in Rome for the treatment of diseases of women and numbered among his clientele patients from over the entire United States. In 1891, he established the Martha Battey Hospital, a benevolent institution for town, country and railway patients. It has been a success from the beginning, and is patronized by the Marine Hospital Service.

Dr. Battey was, at one time, professor of obstetrics in the Atlanta Medical College and, at the same time, editor of the *Atlanta Medical and Surgical Journal.* He held all the positions of honor that could be accorded him by the State Medical Association and was an active member of a large number of medical organizations in this country and abroad. His contributions to the medical press were numerous and valuable.

In 1889, the degree of LL. D. was conferred upon him by the Jefferson Medical College of Philadelphia.

Dr. Battey married, in 1849, to Miss Mary B. Smith, who bore him nine children, eight of whom are now living.

Dr. Battey was not only highly esteemed for his professional skill and attainments, but was loved and respected for his admirable qualities of heart. His life was long and useful, and his death Christian and triumphant.

### PHILADELPHIA ACADEMY OF SURGERY.

### THE SAMUEL D. GROSS PRIZE.

The Second Quinquennial Prize of one thousand dollars, under the will of the late Samuel D. Gross, M. D., will be awarded January 1st, 1900.

The conditions annexed by the testator are that the prize "Shall be awarded every five years to the writer of the best original essay, not exceeding one hundred and fifty printed pages, octavo, in length, illustrative of some subject in Surgical Pathology or Surgical Practice, founded upon original investigations, the candidates for the prize to be American citizens.

It is expressly stipulated that the successful competitor who receives the prize, shall publish his essay in book form, and that he shall deposit one copy of the work in the Samuel D. Gross Library of the Philadelphia Academy of Surgery.

The essays, which must be written by a single author in the English language, should be sent to Dr. J. Ewing Mears, 1429 Walnut Street, Philadelphia, before January 1st, 1900.

Each essay must be distinguished by a motto and accompanied by a sealed envelope bearing the same motto and containing the name and address of the writer. No envelope will be opened except that which accompanies the successful essay. The committee will return the unsuccessful essays if reclaimed by their respective writers or their agents within one year.

The committee reserves the right to make no award if the essays submitted are not considered worthy of the prize.

---

DR. LOUIS H. JONES, who has been so long editorially associated with this journal, has purchased a half interest in it and will, in the future, assume the business management.

Dr. Jones' long experience, combined with his conspicuous business capacity, renders him in every way eminently fitted for the task of safely conducting the SOUTHERN MEDICAL RECORD through the shoals and breakers of medical journalism. Hereafter, all communications relating to the business department of this journal must be directed to him, in person, in order to avoid confusion and delay.

